# Personalized Support in Hereditary Breast and Ovarian Cancer After Genetic Counseling by the Chatbot-Based GENIE Mobile App: Proof-of-Concept Wizard of Oz Study

**DOI:** 10.2196/69115

**Published:** 2025-06-05

**Authors:** Dominik Wolff, Thomas Kupka, Chiara Reichert, Nils Ammon, Steffen Oeltze-Jafra, Beate Vajen

**Affiliations:** 1 Peter L. Reichertz Institute for Medical Informatics of TU Braunschweig and Hannover Medical School, Hannover Medical School, Hanover, Germany; 2 Department for Human Genetics, Hannover Medical School, Carl-Neuberg-Str. 1, Hanover, 30625, Germany, 49 51153280831

**Keywords:** HBOC, hereditary breast and ovarian cancer, Wizard of Oz study, mobile health, evaluation, hereditary diseases

## Abstract

**Background:**

The primary aim of genetic counseling at a human genetics center is to empower individuals at risk for hereditary diseases to make informed decisions regarding their health. In Germany, genetic counseling sessions typically last approximately 1 hour and provide highly personalized information by a specialist in human genetics. Despite this, many counselees report a need for additional support following the counseling session.

**Objective:**

This study introduces GENIE, a chatbot-based mobile app designed to assist individuals in the postcounseling phase, with a focus on hereditary breast and ovarian cancer. GENIE delivers expert-curated, personalized information tailored to the user’s health and family circumstances. The content is presented through predefined dialogs between the user and the mobile assistant, aiming to extend the benefits of genetic counseling beyond the initial session.

**Methods:**

A Wizard of Oz study was conducted to evaluate a functional prototype of GENIE. A total of 6 patients with breast cancer, at least 2 years postdiagnosis, participated in the study. Participants were given access to the app for a minimum of 1 week. The evaluation was based on their interaction with GENIE, which was personalized using the details of a fictitious patient. Data collection included semistructured interviews and a 45-item questionnaire to assess usability and content quality.

**Results:**

The analysis of the interview and questionnaire data indicated high usability for GENIE, with a mean System Usability Score of 75.33 (SD 4.13). In total, 5 of the 6 participants used the app daily; 3 participants were willing to pay between US $5 and US $45 as a single purchase, while the other 3 participants agreed that the app should be free for the user and the costs should be directly covered by health insurance. Still, opinions on the app’s appeal were divided. The layout was seen as moderately professional, a bit crowded, and slightly uninspiring. Nevertheless, participants highlighted the credibility and relevance of the content, noting its alignment with the fictitious patient’s scenario. However, areas for improvement were identified, particularly concerning the app’s design. All participants would recommend the app to other affected persons.

**Conclusions:**

The findings suggest that a mobile app like GENIE can provide valuable support to individuals in the postcounseling phase of genetic services. GENIE offers distinct advantages over large language models, as the information it provides is carefully curated by human experts, minimizing the risk of inaccuracies or hallucinations and significantly enhancing the system’s credibility. This study highlights the need to involve the user group as early as possible in the development of a digital health app. Future work will focus on the implementation of a comprehensive personalization engine, redesign of the user interface, and the execution of a large-scale, 2-arm randomized intervention study to validate GENIE’s effectiveness.

## Introduction

### Background

Breast and ovarian cancers rank among the most common types of cancer in low-income countries [1]. It is estimated that 5%‐10% of all patients with breast cancer and up to 25% of all patients with ovarian cancer exhibit a monogenic predisposition to breast and ovarian cancer [2,3]. Pathogenic variants in specific genes substantially elevate the risk for these cancer types and can be inherited in an autosomal dominant manner, thereby posing a risk to offspring. Female carriers of pathogenic variants in BRCA1 or BRCA2—both high-penetrance genes associated with hereditary breast and ovarian cancer (HBOC)—face a lifetime risk of 50%‐80% for breast cancer, a 60% risk for contralateral breast cancer, and up to 40% for ovarian cancer [1,4,5]. Male carriers of pathogenic BRCA variants have a lower, yet significantly increased, risk of developing breast cancer, estimated between 1% and 7% [6].

Genetic counseling at a recognized human genetics center is a crucial aspect of patient care in Germany, designed to identify individuals at risk for hereditary diseases and provide them with early support. The primary objective of genetic counseling is to empower patients to make informed medical decisions. During the counseling session, patients are assessed for their eligibility for genetic testing of multiple genes associated with HBOC and receive comprehensive, risk-specific information.

In Germany, individuals concerned about a family history of cancer can access genetic counseling. For healthy individuals, genetic counseling is a mandatory prerequisite for genetic testing, as stipulated by the German Genetic Diagnostics Act [7]. Both statutory and private health insurance providers cover the costs of the consultation. Typically, a genetic counseling session lasts approximately 1 hour and is conducted by a medical doctor specialized in human genetics.

The counseling process is highly individualized, taking into account the patient’s family history and disease status. For instance, an individual diagnosed with cancer will receive information tailored to treatment options, while a healthy individual with an elevated familial risk may require different guidance. However, the amount of information presented during these sessions can be overwhelming, especially for individuals in high-stress situations, such as immediately following a cancer diagnosis [8]. In a qualitative study, the authors deciphered the needs of patients in the process of genetic counseling and asked how a future mobile assistant could address them [9]. Patients noted that they need (1) support in the time following genetic counseling, but also (2) in the time before genetic counseling by collecting their own and familial medical information, (3) contact options to support services, (4) patient-friendly medical information, and (5) administration-related assistance in a support app. These demands coincide with Germany’s Digitalization Strategy for Health and Care, which has one focus on the establishment of people-centric, digitally assisted, cross-sectoral, and cross-professional health care processes [10]. One aim of this strategy is to establish need-based digital assistance and support.

In Germany, there are currently two reliable apps, PINK! Coach and Untire, known as “Digital Health Applications (DiGA),” available to support patients with breast cancer with therapy-related concerns [11]. However, there is currently no app available to support HBOC patients during the genetic counseling process in Germany. Talwar et al [12] have analyzed the characteristics and qualities of genetic mobile apps in English, finding that the majority had reference or resource features with general information about genetics and genetic testing (95.5%), targeted mostly health professional students (86.4%), but did not focus on specific diseases (78.5%). Only 21.6% of the apps were developed by reliable or authoritative agencies. Gasteiger et al [13] have reached a similar conclusion when analyzing patient-oriented genetic and genomic mobile apps in the United Kingdom in 2022. In this study, it became evident that only a few high-quality, genetic patient-oriented apps were available in the United Kingdom. They have emphasized the need for more accessible, culturally sensitive, evidence-based apps to improve genetic literacy within patient populations and specific communities, for example, users for whom English is not their native language.

In this paper, we introduce GENIE, a mobile app designed to support individuals both during and after genetic counseling for HBOC. Additionally, we present the results of a proof-of-principle Wizard of Oz study conducted with 6 patients with HBOC who had completed the acute diagnostic and therapy phase, with their initial diagnosis dating back at least 2 years.

### The GENIE Mobile App

The GENIE app is structured in three areas (Compendium, GENIE, and My Profile), which are based on the needs of those receiving human genetic counseling as determined in a prior survey study [9]. We mainly focused on the need for support in the time following genetic counseling by delivering personalized information, and the need for patient-friendly language in communicating medical information. The texts were created taking the Vienna Formula for factual texts into consideration [14]. Average sentence length, word length, and complexity were minimized.

**Figure 1.**
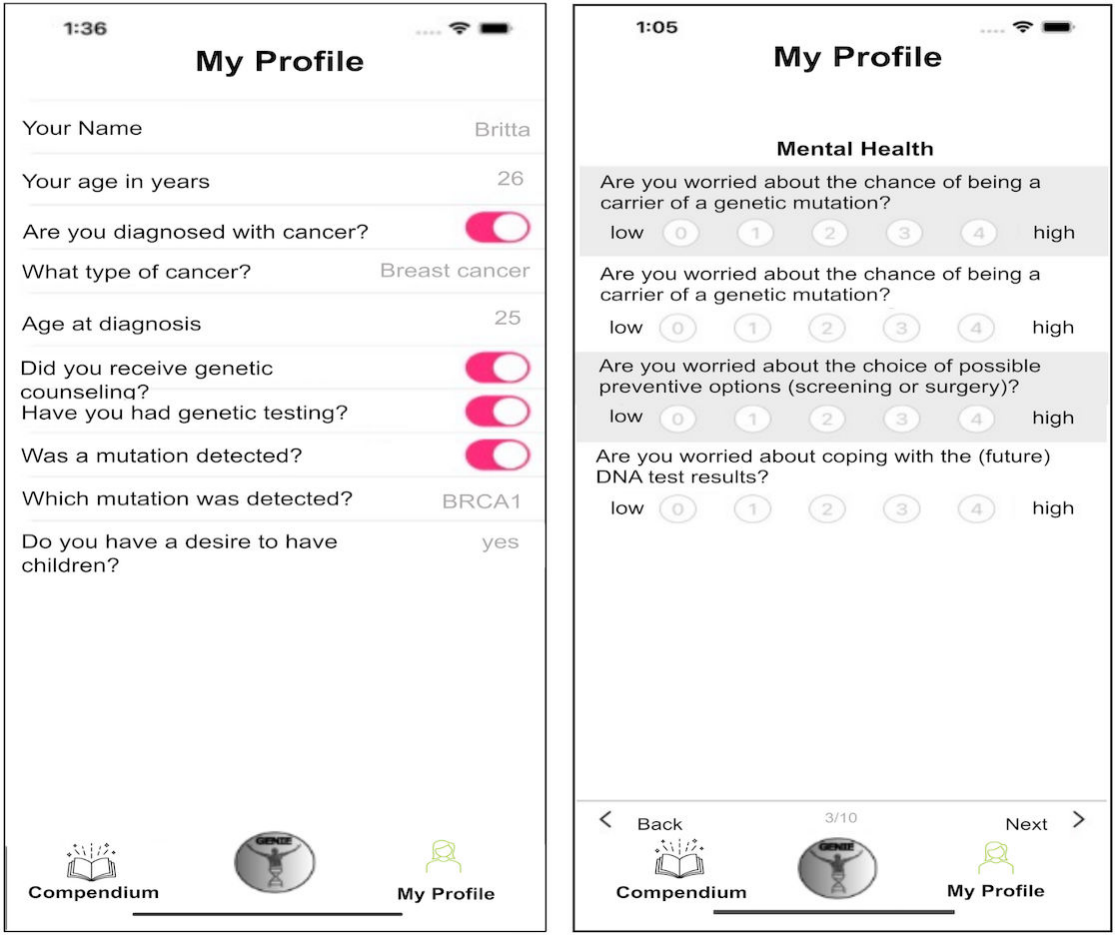
Screenshot of My Profile area in the GENIE app. Users can enter data regarding the user’s diagnosis and counseling status, physical, and psychological condition. These data are needed for personalization.

When starting the app, the user is greeted by the personal assistant GENIE. At the first start of the app, the user is introduced to the app’s features in the form of a tutorial and asked to provide information in the My Profile area (Figure 1).

**Figure 2.**
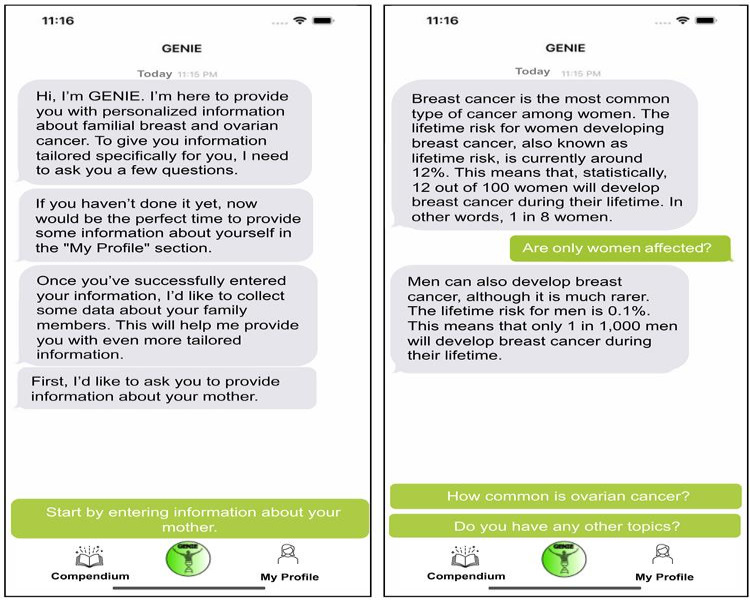
Screenshot of the GENIE area in the GENIE app. During the first start of the GENIE mobile app, the user is greeted by the artificial personal assistant. By briefly introducing its aim and different areas, the visualization is kept as simple as possible for the user (left). The personal assistant starts a conversation about information personalized to the situation of the user. The information is prestructured as a chat, and the user can ask predefined questions regarding the topic (right).

Here, information regarding the user’s specific situation is collected, comprising general, counseling-process-related, and disease-status-related information. The collection is based on a mix of validated assessment tools [15,16] and general questions. This information is then used to select topics personalized to the user. Daily, 5 topics are selected and presented in the GENIE area (Figure 2), which is named after our intelligent agent.

The idea behind the tailored information provision is to minimize excessive information loads. The app’s GENIE area is based on a prestructured dialog and adapts the design of widespread short messaging apps in the form of a chat. Chatting with one’s personal assistant aims to give a feeling of accompaniment and increase adherence. Instead of typing their own questions or messages, the personal assistant initiates the conversation on the selected topics. The user then selects predefined answers regarding whether the topic is interesting and should be deepened by asking a predefined question or whether to move on to the next topic. For each topic, several consecutive in-depth questions are available. After these, the user is directed by a link in the chat to the Compendium area of the app, where more information on this topic exists. After finishing all 5 daily topics, the assistant says goodbye and states that it will provide new topics tomorrow.

**Figure 3.**
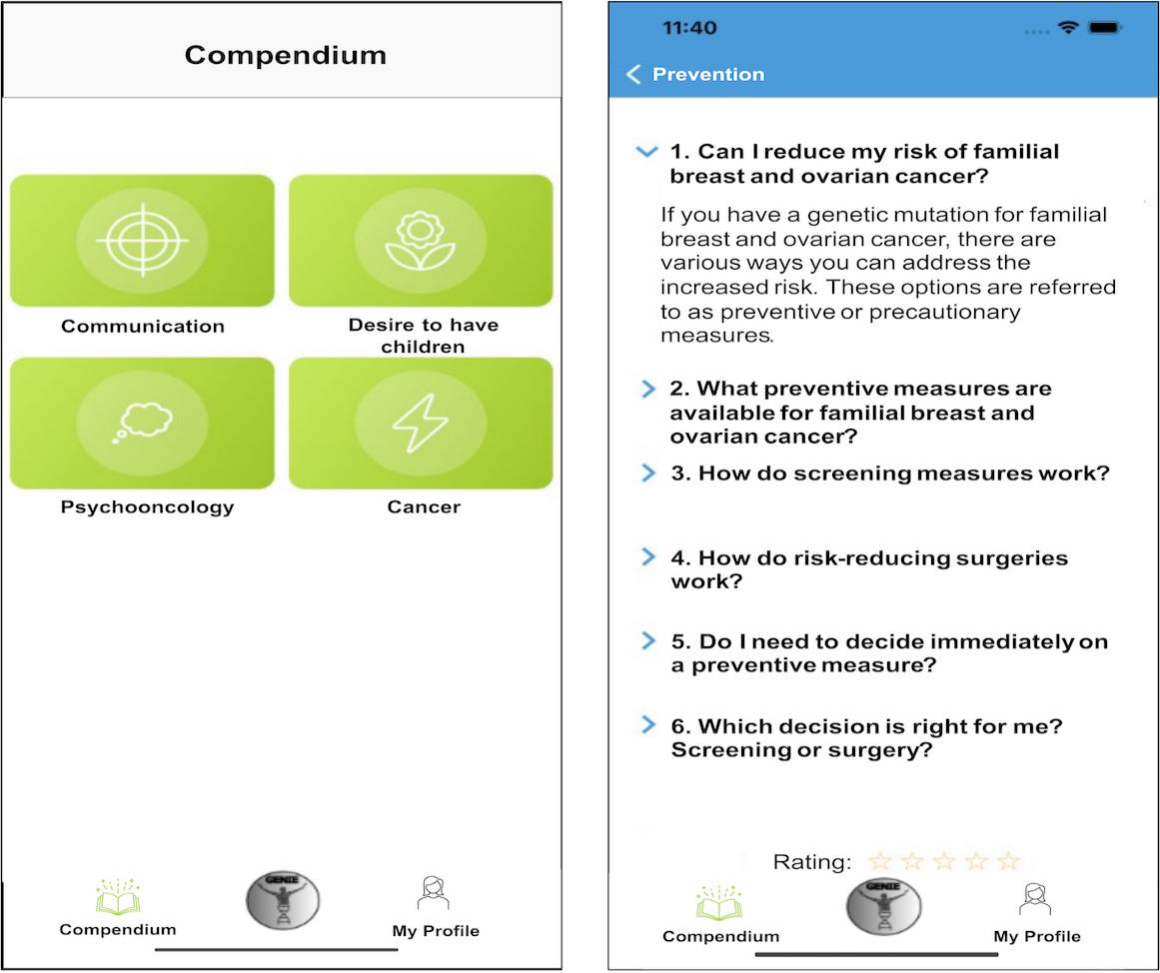
Screenshot of the Compendium area in the GENIE app. The area is structured along the main topics: communication, family planning, psycho-oncology, and disease (left). The individual topics in the Compendium are structured by the questions asked in the dialog. On the bottom, the user can rate the topic (right).

Furthermore, in the Compendium area, the user has unrestricted access to all possible topics the personal assistant can select and provide in the dialog area. This means that besides the personalized topics, additional topics that are potentially not relevant for the specific user can be assessed. Overall, topics are structured by content with communication, family planning, psycho-oncology, and cancer being the main structure (Figure 3).

Each individual topic is structured according to questions similar to the dialog. Furthermore, the user can provide a rating for each topic on a 5-star scale.

For the proof-of-principle presented in the study below, we chose a Wizard of Oz study setting, in which 2 human experts created a fictitious character with specific attributions by hand. In the future, a 2-component artificial intelligence (AI) system will select topics from the corpus of the app based on the data entered in the My Profile area. On the one side, explicit expert knowledge will be formalized as an ontology, and on the other side, tacit knowledge will be implemented based on the scoring system proposed by Wolff et al [17].

We plan to further extend the functionality with the possibility to create a pedigree before genetic counseling and an area to upload administration-related components such as medical reports or direct connection to electronic health records.

## Methods

### Study Design

A partially functional prototype of the GENIE support app was evaluated in the form of a Wizard of Oz study in order to assess user satisfaction. In a Wizard of Oz study, participants believe to interact with an intelligent computer agent, often via a dialog, while the system’s intelligence is mocked by a human [18]. The interaction can be direct, more precise, where the participant directly chats with a human, or indirect, which means that the interaction is mostly prestructured. The methodology was successfully applied to telephone information services such as telephone directories, flight or train information, and reservation services. Recently, the Wizard of Oz study design has been used in user experience studies [19-21]. In this study, two nonfunctional parts, the AI-based personalization of topics and the entering of own information in the My Profile area, existed. Instead, a fictitious profile was used for personalization, whose data were deposited in the My Profile area. For simulating GENIE’s personalization, two experts in human genetics used this profile description for selecting and ordering topics from the corpus of the GENIE app, which can be found in Multimedia Appendix 1. The experts’ ordering was used to simulate the intelligent behavior of GENIE. The used profile was provided to the participants as text in the German language. An English translation follows:

Andrea is 35 years old and was diagnosed with breast cancer some weeks ago. She just finished the genetic counseling session. A genetic test revealed a pathogenic mutation in the BRCA2 gene. Her mother was diagnosed with cancer when she was 40 years old and died shortly after the diagnosis at 41 years due to the disease. Her grandmother on her mother’s side also died of breast cancer but was already 75 years old when she was diagnosed. Her maternal grandfather died of a stroke at 83. Her father is 65 and is doing well apart from a slightly high blood pressure. Andrea’s paternal grandparents have already passed away and lived to a high age. They were both 92 years old. Andrea herself has a son (5 y) and a daughter (7 y). She has a very good relationship with her 2-year-older brother and his 15-year-old son.

Generally, Andrea is a cautious and anxious woman. The cancer diagnosis weighs heavily on her, and she is worried about the future. As a teenager, she felt depressed for several months after the breakup with her first boyfriend. Since then, she has been frightened of being abandoned in her relationships. Due to the diagnosis, Andrea is frightened that her partner Paul might no longer find her attractive, in case of a surgery. She does not know how to communicate the test results to her children. She believes that they are too small to comprehend the situation. Andrea wishes for a third child but is unsure if it is possible with the diagnosis of HBOC. She feels insecure regarding the planning of her family. Therefore, she wishes for more information regarding the preventive options mentioned by the human geneticist during the consultation. She missed that in the conversation due to the huge amount of information explained to her. She would also like to have more information about her breast cancer treatment, as the doctors never really explained it clearly. In view of the overall situation, Andrea would like to receive professional help in the future to cope with her diagnosis psychologically.

Overall, the iCHECK-DH guideline was used for reporting on the GENIE digital health implementation, where applicable in this publication.

### Sample

A total of 6 patients with HBOC evaluated the app after their acute diagnostic and therapy phase. The patient eligibility criteria for the study were: (1) diagnosed HBOC, (2) undertook human genetic counseling, (3) time from first diagnosis at least 2 years (this time gap was required by the ethics committee of Hannover Medical School for the proof of principle study), (4) at least 18 years old, and (5) sufficient German language skills. Recruited participants were given access to the GENIE app for at least a week on a provided iPhone in 2023. Sociodemographic variables were pseudonymized. The patients were selected purposively. All patients presented at the Department of Human Genetics for genetic counseling and testing between 2017 and 2021 and had prior experience in using an iPhone.

The participants’ characteristics are shown in Table 1.

In total, 4 participants had a pathogenic variant in BRCA1, and 2 participants had a pathogenic variant in BRCA2. Of the 6 participants, 4 participants had breast cancer. The participants have no background of immigration, few siblings, and few to no children. They are at the lower end of the middle age range (mean 38.7, SD 8.5 years). In total, 2 participants had a lower level of education (general certificate of upper secondary education or below), and 4 participants were highly educated (at least Abitur). All participants were at work during the testing period.

**Table 1.** Characteristics of the participants evaluating the GENIE app^a^.

ID	Age (years)	Demographics of the center of life	Highest level of education	Immigration background	Number of siblings (f/m)	Number of children (f/m)	Diagnosis	Date of initial diagnosis
1	46	Metropolis	University	No	0/2	0/1	HBOC^b^	June 2020
2	33	Metropolis	University	No	1/0	0/0	HBOC	August 2020
3	40	—^c^	Lower secondary school leaving certificate	No	1/1	0/2	HBOC, breast cancer	February 2020
4	25	Small city	Abitur	No	0/1	0/0	HBOC, breast cancer	May 2021
5	40	Medium-sized city	Secondary school leaving certificate	No	0/1	0/0	HBOC, breast cancer	August 2020
6	48	Small city	Abitur	No	6/1	0/0	HBOC, breast cancer	February 2020

a All patients presented at the Department of Human Genetics at Hannover Medical School for genetic counseling and testing between 2017 and 2021 and were recruited via the BRCA network, a nationwide support network for patients with breast cancer in Germany. All patients had the diagnosis of HBOC.

b HBOC: hereditary breast and ovarian cancer.

c Not applicable.

### Evaluation

The evaluation was divided into 2 parts.

#### Quantitative Study to Evaluate the GENIE App

A 45-item questionnaire in the German language was provided to the participants after the testing of the GENIE mobile app. The questionnaire is structured into the categories: first impression, contents, app layout, usability, use, and willingness to pay. The categories, first impression, contents*,* and usability consist of an excerpt from Thielsch et al, 2017 [22] and the System Usability Scale by Brooke [23], respectively. The categories, first impression, contents, and app layout consist of a 7-item Likert scale, while the System Usability Scale uses a 5-item Likert scale. The use and willingness to pay are each a binary yes-no question and a free text field for the provision of reasons. The data were analyzed descriptively and visually using a bar plot in Python (Python Software Foundation). Therefore, ratings of negatively formulated questions, such as “the colors do not match,” were transformed to the positive scale by subtracting the negative formulated rating from the scale’s maximum and adding 1 (pos=maxRating–negative+1). The questionnaires, along with the participants’ consent forms, are stored in a fireproof cabinet at the Institute of Human Genetics and will be kept for 10 years.

#### Qualitative Study to Evaluate the GENIE App

The qualitative study was conducted in the summer of 2023 in Hannover, Germany, to explore the satisfaction of the participants with the GENIE app. All participants were interviewed 2 to 3 weeks after testing the GENIE app. The interviews were performed by NA. NA is a fifth-year medical student at the University Medical Center Freiburg and tested the GENIE app within his PhD study. Due to valuable experience in qualitative analyses in the field of human genetics, BV (research associate at the Department of Human Genetics) provided close supervision of NA. No patient knew the interviewer before. Interviews were conducted as a video call (face-to-face) at home or in the office of the Department of Human Genetics and took place between the researcher and the patient only. The interviewer introduced himself as a medical doctoral student with a particular interest in supporting patients in the process of genetic counseling through mobile support apps. A semistructured interview guide was used, which roughly defined the structure and topics of the interview with open questions, but still allowed sufficient flexibility for new topics.

1. How did you find the layout and structure of the app?
2. How did you find the different sections of the app?
3. How did you find the design of the app?
4. How did you find the information density, clarity, and text length?
5. How did you find the suitability of the texts for Andrea?
6. Did you find the content trustworthy?
7. Did you feel like you were building a relationship with GENIE?
8. Would you recommend the app?

The interviews were audio recorded.

### Data Analysis

The audio files of the interviews were transcribed and digitized. Transcription was done according to predefined rules following Kuckartz and Rädiker [24] and Dresing et al [25]. The interviews were evaluated according to structuring content analysis, a subform of qualitative content analysis according to Kuckartz et al [24]. For the evaluation, the software MAXQDA (version 2022; VERBI software) was used. All interviews were included in the analysis.

The following quality criteria, according to Mayring [26], were met during the evaluation:

1. Intercoder agreement—to ensure objectivity, a second person (BV) was involved in the coding process. In case of ambiguity, the researchers discussed the classification of the data into the appropriate categories. The defined category system ensured an intersubjectively comprehensible analysis or category assignment.
2. Intracoder agreement was assured by NA through repeated coding at a later point in time.
3. Procedural documentation—the process of data collection and data analysis was documented in a way that was transparent and comprehensible to others.
4. Rule guidance—the process of data collection and data analysis was systematic and rule-governed (interview protocols, transcription rules, coding rules)
5. Proximity to the object—the data collection took place in the everyday life of the interviewees and was based on the problems of the interviewees.

### Ethical Considerations

The study was performed in accordance with the ethical standards of the responsible committee on human experimentation (institutional and national) and with the Helsinki Declaration of 1975, as revised in 2000. The study was approved by the Ethics Committee of Hannover Medical School (10573_BO_K_2022). Informed written consent was obtained from all patients to be included in the study. Participants were informed that participation was voluntary, and that they could withdraw at any time. Furthermore, they were informed about the right to access, rectify, restrict, and delete their data during the study. Patients were informed that nonparticipation would not result in any disadvantages for them. The participants gave their consent for the interview to be recorded and transcribed. The audio files and the data from the questionnaire were stored on a server at the Hannover Medical School (MHH) and are safeguarded according to the principles of Good Scientific Practice of the German Research Foundation. All data will be stored for 10 years on the servers or in locked cabinets at the Institute of Human Genetics at MHH, after which it will be deleted. The interview transcripts and questionnaire data were pseudonymized. In case of withdrawal, there is a list of participants that has been stored separately to allow the identification of individual data. Only project staff from MHH have access to the data. MHH operates in accordance with the provisions of the General Data Protection Regulation, the Federal Data Protection Act of May 25, 2018, and the Lower Saxony Data Protection and Freedom of Information Act of May 16, 2018. No data were shared with third parties. Participants were compensated with US $112 for their efforts. Participants were recruited via email via the BRCA Network, a nationwide support network for patients with breast cancer in Germany. All patients presented at the Department of Human Genetics for genetic counseling and testing between 2017 and 2021.

## Results

In the following, we will present the quantitative results from the testing, followed by the qualitative data gathered from the interviews.

### Results of the Quantitative Analysis

**Figure 4.**
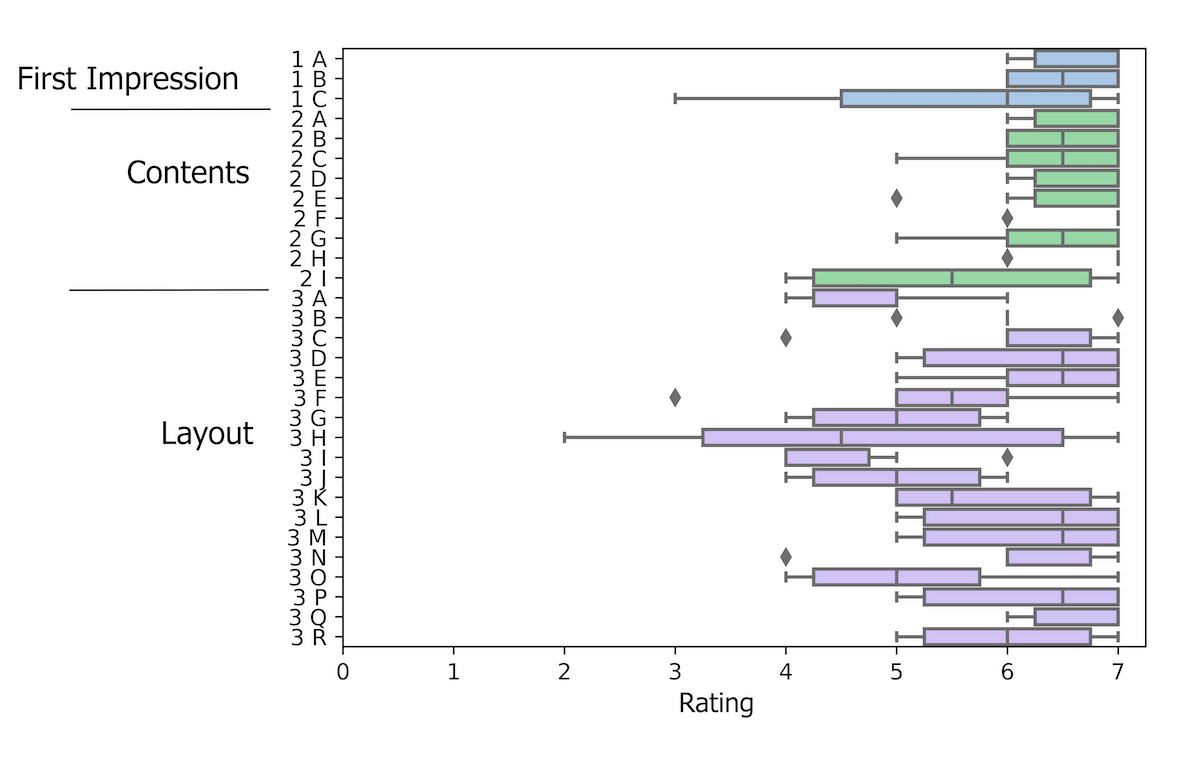
Participants’ ratings by categories and questions (y-axis) with rating scales according to the methods section (x-axis). A total of 6 participants tested the GENIE app for 1 week and rated the first impression by answering 3, the contents by answering 9, and the layout of the GENIE app by answering 18 questions on a 7-item Likert scale, with 1 being the lower (negative) and 7 being the upper (positive) end of the scale. First impression: 4.29 (SD 2.09); contents: 5.01 (SD 1.61); layout: 4.48 (SD 1.36). A glossary of the abbreviations in the y-axis to the corresponding questionnaire items can be found in Multimedia Appendix 1.

All participants finished the questionnaire. The questions are provided in Multimedia Appendix 1. Overall, the participants’ first impression was that the app is usable (1B), which is further supported by a high mean System Usability Score of 75.33 (SD 4.13). On the other hand, users were discordant regarding the app’s appeal (1C). The app’s content was interesting on the first impression (1A), as well as after using the app (2A-H). Some participants even wanted to save or print out some of the topics provided (2I). The layout of the app achieved a mean rating of 4.48 (SD 1.36) points on the 7-item Likert scale. On a single-item level, the participants found that the layout is only moderately professional (3O), a bit crowded (3A), and slightly uninspiring (3G), but also pleasantly organized (3C). A complete overview of the results can be found in Figure 4.

Further, 5 of 6 participants answered that they were using the app daily during the evaluation. Regarding the willingness to pay for the mobile app, 3 participants would pay amounts between US $5 and US $45 as a single purchase, while the other 3 expressed that the app should be free for users and financed by health insurance or pharmacy companies.

### Results of the Qualitative Analysis

The structured interviews primarily addressed the following five points: (1) clarity and text length, (2) credibility, (3) building a relationship level through dialog, (4) personalization of the dialog, and (5) recommendation of the GENIE app.

#### Clarity and Text Length

All participants rated the clarity of the texts and the text length as very good. It was noted that it is very important for the participants that the texts are also understandable for medical laypersons, which is not always the case in doctor consultations and on the internet.

*Very easy to understand. Very, very simple to read. Very understandable. This way, you can process multiple topics at once or combine them into one because, when talking to doctors or reading elsewhere, it’s often described in very technical terms, which I find to be very specialized. But in the app, I found it very, very pleasant*.

[Participant 4]

#### Credibility

The GENIE app is characterized by evidence-based texts, and source references are stated at the end of the texts. Therefore, we were interested in whether users considered the source references important. Some users immediately noticed the source references and rated them positively.

*I actually find it very useful and very good, especially when you already have a few points in an area where you have read up, and then realize: Okay, I want to delve deeper into this now. Instead of sitting down and googling everything like crazy, I immediately know: Okay, this information comes from here and there. I can continue to educate myself further and explore it in more depth. It’s definitely useful.*

[Participant 5]

#### Building a Relationship Level Through Dialog

Users can access personalized information through the GENIE app in two ways: via a chatbot in the dialog area and via a well-structured knowledge base, such as an encyclopedia. To assess user perceptions of the text delivery strategy, we investigated how the participants experienced these methods. In total, 5 out of 6 participants preferred the dialog area and used the knowledge base only minimally. One participant noted limited use of the dialog feature and reported that she primarily engaged with the texts in the knowledge base, commending their clarity and organization. Additionally, some participants who favored the dialog area developed a sense of rapport with the app, feeling as if they were conversing with a health care professional or another individual with a similar condition. This manifested as an unplanned yet beneficial outcome.

*Yes, sometimes I had the feeling as if the person was sitting next to me, explaining things to me or reading something aloud, or asking me questions, and I would answer, or I would ask and they would respond. It felt like, well, like a doctor or a medical student who is also engaging with the topic themselves.*

[Participant 2]

*It’s very personal, and that makes it very pleasant, especially in a health context, it’s good to have someone by your side. I know this from personal experience as well. It’s nice to have someone who gives you the feeling that they’ve been through it themselves and can help you with a lot of knowledge. And that’s exactly how it is (...) with the app. This personal touch is a great idea.*

[Participant 4]

#### Personalization of the Dialogs

One of the key features of the GENIE app is its high level of personalization. The participants were asked to assess how well the content of the texts matched Andrea’s situation. All participants stated that the suggested texts fit Andrea’s described situation very well.

*Yes, yes. It was perfect, exactly one-to-one. Nothing was missing. I would say it fit exactly with what Andrea had mentioned about her situation, and the information that came to Andrea was wonderful.*

[Participant 2]

### Recommendation

At the end of the interview, the participants were asked if they would recommend the app. All participants said they would recommend the app to others affected. In total, 2 participants mentioned that they would have liked to use this app themselves when they received the diagnosis of hereditary breast cancer.

*I found the app very good, and it was explained superbly. I would recommend it to anyone who has issues with this genetic mutation or human genetics. I would say: Try it out, maybe you’ll understand it better, and it’s explained in a very simple way.*

[Participant 2]

## Discussion

### Principal Findings

In this study, we were able to demonstrate the benefits of the autonomous mobile support GENIE. The app has the potential to support women during the phase of genetic counseling in the contemplated areas. The qualitative data from the interviews and the data from the questionnaires show the app’s high usability and high quality of its content. Still, the design needs to be revised. Overall, the time and location-independent use of the app, while personalizing the topics, satisfied the participants. The simulation of a personal assistant in the genetic counseling process was successful. The only functionality not used was the star rating component. Here, an additional message by the assistant hinting at it could be helpful. In the future, the star rating could be used to further refine the personalization of topics.

Patients diagnosed with HBOC have diverse needs. This group includes women who have already been diagnosed with breast and/or ovarian cancer, as well as healthy women whose cancer risk is merely elevated. Metcalfe et al [27] observed that women with a prior cancer diagnosis indicated a need for more information related to cancer treatment compared to women without a cancer history. Another study demonstrated that women with pathogenic variants in moderate-risk genes for HBOC have distinct informational needs compared to those with variants in high-risk genes [28]. Consequently, Henneman et al [29] suggested that the genetic counseling process should be tailored to meet individual demands and consider the expectations and pre-existing lay knowledge of the counselee. In this evaluation study, we were able to show that most participants preferred the tailored information delivered by the chatbot over the Compendium.

One of the key features is the credibility and clarity of the provided information. The patients reported that the provision of references in the Compendium enhanced the credibility. The high level of credibility and clarity of information provided via the GENIE app is achieved by expert manual curation. However, this process is time-consuming and holds the risk of information not being up to date within the revision’s time delay. In the future, we will explore the use of large language models (LLMs) to (partially) automate this process. The provision of information in the form of a dialog resulted in patients perceiving the interaction as if they were speaking with a certified specialist in human genetics. Credibility and perceived competence are well-known factors influencing patient adherence [30,31]. Furthermore, the literature already highlights a mismatch between the information provided during counseling sessions and patients’ expectations regarding the desired content, usefulness, and comprehensibility [32-34]. Factors such as the health literacy level of counselees and the appropriateness of language used for lower levels have been identified to have a significant influence on genetic counseling. Additionally, it is widely recognized that verbal medical information provided by health care practitioners, particularly genetic information, is often not accurately recalled. Research on information recall indicates that up to 80% of medical information given by health care practitioners is forgotten, and about half of the information remembered is incorrect [35]. This impacts not only the patient but also family members who receive incorrect or insufficient information. Furthermore, according to Edwards et al [36], emotional and supportive communication is beneficial for the counselors and prevails as the sole benefit of having more information. Although the personal assistant was perceived as a (human-like) specialist in human genetics by some participants, a study conducted prior to the development of the GENIE app emphasized the importance of human interaction with patients. In that study, patients reported that the interpersonal interaction with the genetic specialists was experienced as particularly positive [9]. The importance of human interaction with patients, in general, was also emphasized. Nonetheless, the use of an autonomous chatbot before and after the counseling session can increase the time available for emotional support during the sessions.

A potential risk of autonomous systems such as GENIE or LLMs like ChatGPT in health-related contexts is the potential increase in so-called cyberchondria, which is characterized by excessive web-based health research and heightened, yet unnecessary, anxiety [37,38]. Multiple aspects of these systems could be associated with cyberchondria: (1) information accessibility, (2) information quality, and (3) credibility. Both GENIE and LLMs serve as low-threshold gateways to information, characterized by easy access and time-independent availability, making repeated and excessive consumption effortless. This, in turn, may potentially foster negative behavior patterns in individuals with cyberchondria.

Nonetheless, GENIE offers several advantages over LLMs. First, the quality of the information is controlled by human experts who manually create and curate the texts provided by the system. Thus, hallucinations, and fictitious or inaccurate information, which can occur in LLMs [39,40], are very unlikely to occur in our agent. In addition, this directly impacts the system’s credibility. For GENIE, scientific information sources are provided, which were used to curate the texts. For LLMs, even in the case of using scientific literature as a corpus, it cannot be ruled out at the moment that fictitious publications would be presented by the system as proof of its statements. The same is true for the appropriateness of information for the users. Moreover, in Germany, the Genetic Diagnostics Act prohibits giving direct advice in the genetic counseling session. To the authors’ knowledge, there is currently no safe possibility to exclude such behavior in LLMs. On the other hand, the manual curation of texts by experts, including web search, selection of scientific publications, and revision of information in the form of prestructured dialogs, is very laborious and may limit the width of topics that can be recommended by the system.

### Limitations

Still, the results of this study are limited by multiple factors. First, the study design as a Wizard of Oz experiment holds the risk that the later system’s personalization engine cannot live up to the high personalization standards provided by the experts. Simulating GENIE’s AI component and relying on an expert’s selection for personalization of the topics holds the risk that the implementation of such a recommender system lacks proper individualization performance. Especially, the formalization of expert knowledge holds the risk of being lossy. Still, in the case of a working personalization system and its use in the real world, the system would be robust regarding changes in the user’s profile, which may evolve over time. By updating the profile information in the My Profile area of the app, the system would use the updated information to adapt the selection of topics. Second, the results presented are based on a single fictitious case study, making it possible that the system’s topic recommendation fails for other cases. Other limitations are found in the limited study cohort. The number of 6 participants seems rather low. Still, for usability testing, 5 participants are typically able to find the majority of errors [41]. On the other hand, the participants were not in the acute phase of genetic counseling, making it difficult to analyze if the app would stress or even harm participants in this phase. Here, some participants expressed that they wished to have had the app during their acute phase. Although all participants had prior experience using an iPhone, wide market coverage is crucial. We are therefore currently in the process of porting the app to Android while reworking the user interface. Furthermore, all participants are female, and thus, gender aspects are not analyzable. However, the main target audience of the GENIE app is women since they are more prone to HBOC than men. To overcome these limitations, we plan to conduct a two-arm, randomized intervention study with 240 participants at the Institute of Human Genetics at Hannover Medical School, in cooperation with the Center for Familial Breast and Ovarian Cancer and the Department of Biometry at Hannover Medical School. Here, a fully functional version of the GENIE app will be used to provide scientific evidence of the effectiveness of the GENIE app in delivering a positive health care impact for patients with HBOC.

### Conclusions

Widespread use of GENIE has the potential to fill existing gaps in the current landscape of mobile solutions for hereditary diseases. Patients reported in interviews that they felt abandoned after genetic counseling. The study presented here demonstrates that GENIE can provide support to patients in this regard. In the next step, we will implement the topic recommendation system simulated in this study by human expertise via the mentioned approaches, precisely an ontology and the scoring system described in Wolff et al [17], by acquiring and formalizing the expert’s knowledge for topic personalization. Moreover, we are currently revising the mobile app regarding user interfaces and user experience to improve its usability, together with an expert industry partner. Additionally, we will extend the system’s functionality in order to cover further requirements and wishes of patients reported in Ammon et al [9], such as support in creating one’s pedigree before genetic counseling and an area to upload administration-related components such as medical reports or direct connection to electronic health records. Furthermore, the intervention study will help to confirm GENIE’s positive health care impact on patients with HBOC.

## Supplementary material

10.2196/69115 Multimedia Appendix 1 Sorted list of topics relevant to Andrea.

10.2196/69115 Checklist 1 Checklist of iCHECK-DH: Guidelines and Checklist for the Reporting on Digital Health Implementations.

## Abbreviations

AI: artificial intelligence
HBOC: hereditary breast and ovarian cancer
LLM: large language model
MHH: Hannover Medical School

## References

[1] Nielsen FC, van Overeem Hansen T, Sørensen CS (2016). Hereditary breast and ovarian cancer: new genes in confined pathways. Nat Rev Cancer.

[2] Campeau PM, Foulkes WD, Tischkowitz MD (2008). Hereditary breast cancer: new genetic developments, new therapeutic avenues. Hum Genet.

[3] Walsh T, Casadei S, Lee MK (2011). Mutations in 12 genes for inherited ovarian, fallopian tube, and peritoneal carcinoma identified by massively parallel sequencing. Proc Natl Acad Sci U S A.

[4] Goldberg JI, Borgen PI (2006). Breast cancer susceptibility testing: past, present and future. Expert Rev Anticancer Ther.

[5] Antoniou A, Pharoah PDP, Narod S (2003). Average risks of breast and ovarian cancer associated with BRCA1 or BRCA2 mutations detected in case Series unselected for family history: a combined analysis of 22 studies. Am J Hum Genet.

[6] Tai YC, Domchek S, Parmigiani G, Chen S (2007). Breast cancer risk among male BRCA1 and BRCA2 mutation carriers. J Natl Cancer Inst.

[7] Human Genetic Examination Act [Web page in German]. Federal Ministry of Justice and Consumer Protection.

[8] Vajen B, Rosset M, Wallaschek H, Baumann E, Schlegelberger B (2021). Psychological distress and coping ability of women at high risk of hereditary breast and ovarian cancer before undergoing genetic counseling—an exploratory study from Germany. Int J Environ Res Public Health.

[9] Ammon N, Reichert C, Kupka T (2024). Deciphering the needs of patients with hereditary breast and ovarian cancer in the process of genetic counseling to inform the development of a mobile support app: a qualitative study in Germany. J Community Genet.

[10] Digital together: Germany’s digitalisation strategy for health and care. https://www.bundesgesundheitsministerium.de/fileadmin/Dateien/3_Downloads/D/Digitalisierungsstrategie/Germany_s_Digitalisation_Strategy_for_Health_and_Care.pdf.

[11] (2024). DiGA directory [Web page in German]. Federal Institute for Drugs and Medical Devices.

[12] Talwar D, Yeh YL, Chen WJ, Chen LS (2019). Characteristics and quality of genetics and genomics mobile apps: a systematic review. Eur J Hum Genet.

[13] Gasteiger N, Vercell A, Davies A, Dowding D, Khan N, Davies A (2022). Patient-facing genetic and genomic mobile apps in the UK: a systematic review of content, functionality, and quality. J Community Genet.

[14] Bamberger R, Vanecek E (1984). Reading–Understanding–Learning–Writing: The Levels of Difficulty of Texts in the German Language [Book in German].

[15] Tercyak KP, Johnson SB, Roberts SF, Cruz AC (2001). Psychological response to prenatal genetic counseling and amniocentesis. Patient Educ Couns.

[16] Eijzenga W, Bleiker EMA, Hahn DEE (2014). Psychosocial aspects of hereditary cancer (PAHC) questionnaire: development and testing of a screening questionnaire for use in clinical cancer genetics. Psychooncology.

[17] Wolff D, Behrends M, Gerlach M, Kupka T, Marschollek M (2018). Personalized knowledge transfer for caregiving relatives. Stud Health Technol Inform.

[18] Dahlbäck N, Jönsson A, Ahrenberg L (1993). Wizard of Oz studies—why and how. Knowl Based Syst.

[19] Weiss A, Bernhaupt R, Schwaiger D, Altmaninger M, Buchner R, Tscheligi M User experience evaluation with a Wizard of Oz approach: technical and methodological considerations.

[20] Ghosh S, Shah C (2025). Spoken conversational search: evaluating the effect of system clarifications on user experience through Wizard‐of‐Oz study. J Assoc Inf Sci Tech.

[21] Salber D, Coutaz J Applying the Wizard of Oz technique to the study of multimodal systems.

[22] Thielsch MT, Niesenhaus J (2017). The Wiley Blackwell Handbook of the Psychology of the Internet at Work.

[23] Brooke J (1996). Usability Evaluation in Industry.

[24] Kuckartz U, Rädiker S (2022). Qualitative Content Analysis: Methods, Practice, and Computer Support [Book in German].

[25] Dresing T, Pehl T (2018). Practical Guide to Interviews, Transcription & Analysis: Instructions and Rules for Qualitative Researchers [Book in German].

[26] Mayring P (2022). Qualitative Content Analysis: Principles and Techniques [Book in German].

[27] Metcalfe KA, Liede A, Hoodfar E, Scott A, Foulkes WD, Narod SA (2000). An evaluation of needs of female BRCA1 and BRCA2 carriers undergoing genetic counselling. J Med Genet.

[28] Stracke C, Lemmen C, Rhiem K, Schmutzler R, Kautz-Freimuth S, Stock S (2022). Medical knowledge and information needs among women with pathogenic variants in moderate-risk genes for hereditary breast cancer attending genetic counseling at an academic hospital in Germany—a qualitative approach. J Genet Couns.

[29] Henneman L, Timmermans DRM, van der Wal G (2004). Public experiences, knowledge and expectations about medical genetics and the use of genetic information. Community Genet.

[30] Dai Z, MacDorman KF (2018). The doctor’s digital double: how warmth, competence, and animation promote adherence intention. PeerJ Comput Sci.

[31] Polinski JM, Kesselheim AS, Frolkis JP, Wescott P, Allen-Coleman C, Fischer MA (2014). A matter of trust: patient barriers to primary medication adherence. Health Educ Res.

[32] Joseph G, Lee R, Pasick RJ, Guerra C, Schillinger D, Rubin S (2019). Effective communication in the era of precision medicine: a pilot intervention with low health literacy patients to improve genetic counseling communication. Eur J Med Genet.

[33] Kamara D, Weil J, Youngblom J, Guerra C, Joseph G (2018). Cancer counseling of low-income limited English proficient Latina women using medical interpreters: implications for shared decision-making. J Genet Couns.

[34] Cheng JKY, Guerra C, Pasick RJ, Schillinger D, Luce J, Joseph G (2018). Cancer genetic counseling communication with low-income Chinese immigrants. J Community Genet.

[35] Kessels RPC (2003). Patients’ memory for medical information. J R Soc Med.

[36] Edwards A, Gray J, Clarke A (2008). Interventions to improve risk communication in clinical genetics: systematic review. Patient Educ Couns.

[37] Starcevic V, Berle D, Arnáez S (2020). Recent insights into cyberchondria. Curr Psychiatry Rep.

[38] Starcevic V (2017). Cyberchondria: challenges of problematic online searches for health-related information. Psychother Psychosom.

[39] Perković G, Drobnjak A, Botički I Hallucinations in LLMs: understanding and addressing challenges.

[40] Hu R, Zhong J, Ding M, Ma Z, Chen M Evaluation of hallucination and robustness for large language models.

[41] Nielsen J, Landauer TK (1993). A mathematical model of the finding of usability problems.

