# Supplementary material for: Personalized Support in Hereditary Breast and Ovarian Cancer After Genetic Counseling by the Chatbot-Based GENIE Mobile App: Proof-of-Concept Wizard of Oz Study
Source: JMIR Form Res. 2025 Jun 5;9:e69115. doi: 10.2196/69115 (PMC12161161; doi:10.2196/69115)
Supplement: Multimedia Appendix 1 [file formative-v9-e69115-s001.docx]

**Sorted list of topics relevant to Andrea**

• Diagnostics for HBOC with positive gene test

• Aetiology of HBOC

• Risk genes

• Symptoms of HBOC

• Epidemiology

• Therapies of HBOC and PARP-Inhibitors

• Overview of breast cancer therapies

• Therapy: Chemotherapy for breast cancer

• Psycho-oncology and coping

• Social services

• Fear

• Stress

• Relaxation techniques

• Sexuality and emotions

• Coping with the diagnosis HBOC

• Fertility and HBOC

• Family planning with HBOC

• Communication with children: sharing genetic testing results

• Precautionary measures: Overview and decision making

• Precautionary measures: Mammography for high-risk genes

• Precautionary measures: risk reducing operation - Mastectomy

• Precautionary measures: risk reducing operation - Breast reconstruction surgery

• Precautionary measures: risk reducing operation - Oophorectomy with BRCA

• Precautionary measures: risk reducing operation - Consequences of ovarian cancer

• Precautionary measures: risk reducing operation - Hormone replacement therapy for ovarian cancer

**Questionnaire (translated from German)**

1: First impression

A) I find the content of this app interesting.

B) I consider the app to be user-friendly.

C) I find this app visually appealing.

D) I would rate the app overall as (excellent, good, satisfactory, sufficient, poor).

2: Contents

A) The app piques my interest.

B) I like the content of the app.

C) I enjoy reading in this app.

D) The individual sentences are easy to read.

E) The texts provide me with concise and essential information.

F) The language used in the texts is familiar and easy to understand.

G) The information is of high quality.

H) I find the information in the app useful.

I) The content of the app seems so important to me that I would print it out or save it.

3: Layout

A) The layout feels crammed.

B) The layout is easy to grasp.

C) The layout appears well-structured.

D) The app seems too inconsistent.

E) Everything in the app fits together.

F) The design of the app is uninteresting.

G) The layout is original.

H) The design feels uninspired.

I) The layout feels dynamic.

J) The layout is pleasantly versatile.

K) The overall color scheme is attractive.

L) The colors don’t match.

M) The color choices are unsuccessful.

N) The colors have a pleasant effect.

O) The layout is professional.

P) The layout is outdated.

Q) The app appears to be carefully designed.

R) The layout feels aimless.
